# Vision in Flies: Measuring the Attention Span

**DOI:** 10.1371/journal.pone.0148208

**Published:** 2016-02-05

**Authors:** Sebastian Koenig, Reinhard Wolf, Martin Heisenberg

**Affiliations:** Rudolf-Virchow-Center, Joseph-Schneider-Strasse 2, 97080 Würzburg, Germany; Alexander Fleming Biomedical Sciences Research Center, GREECE

## Abstract

A visual stimulus at a particular location of the visual field may elicit a behavior while at the same time equally salient stimuli in other parts do not. This property of visual systems is known as selective visual attention (SVA). The animal is said to have a focus of attention (FoA) which it has shifted to a particular location. Visual attention normally involves an attention span at the location to which the FoA has been shifted. Here the attention span is measured in *Drosophila*. The fly is tethered and hence has its eyes fixed in space. It can shift its FoA internally. This shift is revealed using two simultaneous test stimuli with characteristic responses at their particular locations. In tethered flight a wild type fly keeps its FoA at a certain location for up to 4s. Flies with a mutation in the *radish* gene, that has been suggested to be involved in attention-like mechanisms, display a reduced attention span of only 1s.

## Introduction

Visual stimuli may guide or even trigger an animal's behavior but, inversely, an animal may also actively generate, test or select a visual stimulus to organize its behavior. In this case, the animal may restrict full depth visual processing to the relevant part of the visual field. This property of visual systems is known as selective visual attention (SVA).

SVA has first been described in humans [1] and was subsequently studied also in animals [2–4]. In insects, despite early observations [5,6], very little is known about it [7–12].

In the present study we report on a characteristic property of SVA, the attention span, in the fly *Drosophila*. We measure yaw torque, a motor component of intended flight control, in an individual tethered fly. The fly has its head and as a consequence also its eyes fixed in space. Hence, covert SVA undisturbed by shifts of gaze can readily be studied in this preparation. SVA can be deployed to objects or certain features or to a certain location in space [13]. The latter has been shown for *Drosophila*, which can in this situation restrict its behavioral responses to stimuli in distinct parts of the visual field [6,14]. Visual stimuli at particular locations can elicit characteristic responses. If, repeatedly, two such stimuli are simultaneously displayed at different locations and the fly spontaneously switches between the corresponding responses, the fly is assumed to shift its focus of attention (FoA) to this or that particular location (covert attention, [15]). The concept of SVA in *Drosophila* is strengthened by the observation that the FoA can be attracted by external non-visual [14] and visual stimuli [16].

Humans shift their FoA to a certain location in order to scrutinize what they can see there. The FoA may stay there for some time. Is such an 'attention span' also found in flies? If so, how long does it last? To answer these questions SVA is tested without cueing. Following the nomenclature used in human psychology [17], shifting the FoA via external cues represents a bottom-up modulation of SVA, whereas internal shifts as studied here represent a top-down modulation. Again, two stationary stripes are shown to the fly. To test for the location of the FoA both stripes are suddenly displaced front-to-back. The fly may not respond at all but if it does, it responds to either the left or the right stripe with about equal probability. We now repeat the test and measure the response probabilities again. They are biased towards the side of the first response.

## Results

### Displacement of a stripe may elicit different response patterns

**Fig 1:**
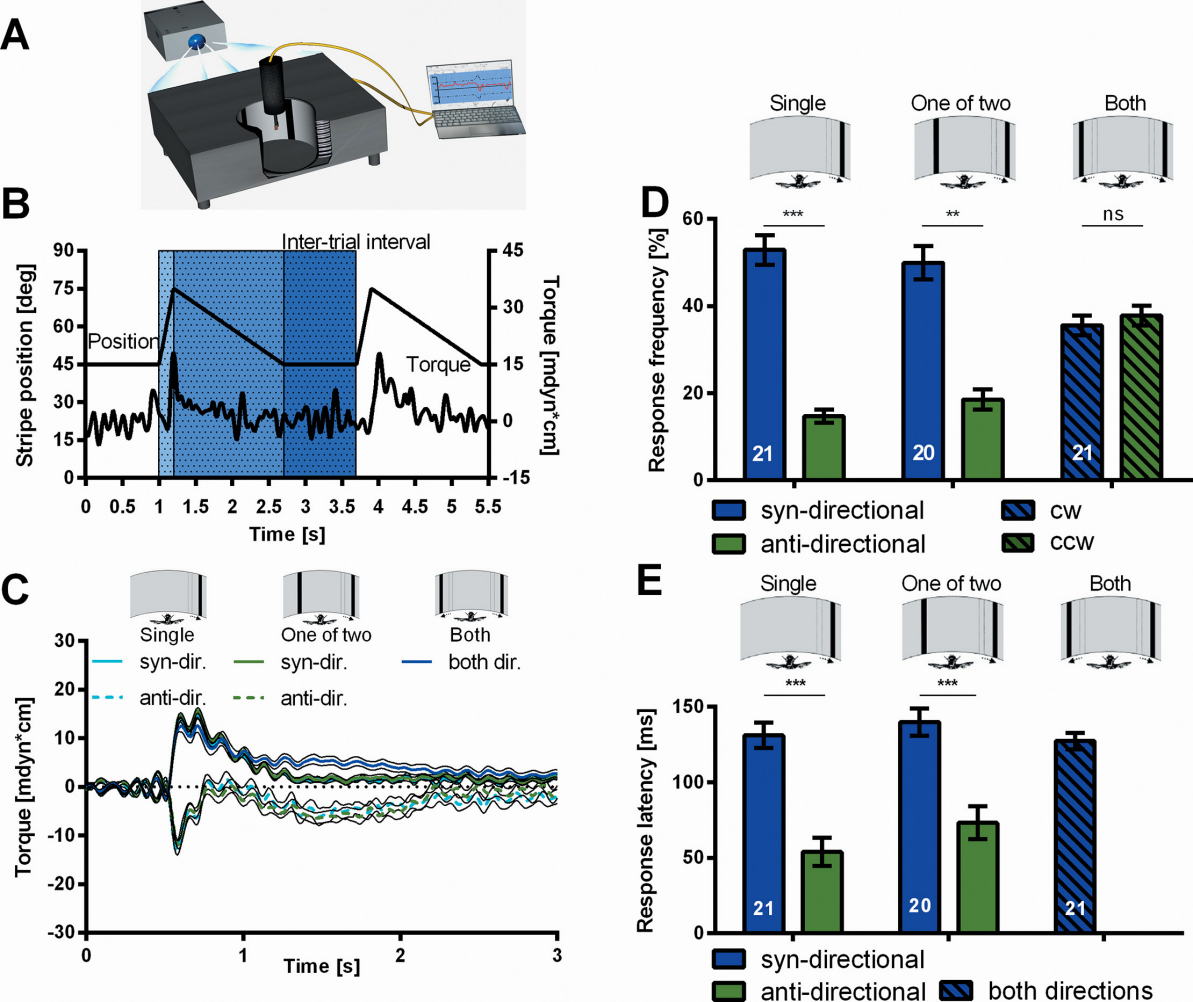
Yaw torque responses to front-to back displacements of one or two stripes. (A) Experimental setup. A single fly is attached to a torque meter and centered in a fiber-optics arena. The visual stimuli are generated in a commercial computer and projected via a commercial projector onto one end of the fiber optics, which then transfers the image onto the inner 360°-surface of the arena. (B) Sequence of stimuli and prototypical torque responses. A single black stripe is displaced front-to-back on white background (Δψ = 30° at 150°/s) and then slowly reset at 20°/s to its initial position. It remains at this position for the duration of the inter-trial interval (ITI) until another displacement starts. In most of the cases the fly responds to the displacement with a phasic modulation of torque with the same polarity as the motion of the stripe (jagged line: torque; smooth line: stripe position). (C) Average CantonS torque traces of the responses to a displacement under three different experimental conditions. Responses to the dispacement of a single stripe (‘Single’, N = 21) can be syn-directional or anti-directional. With a stationary stripe presented in the arena in addition (‘One of two’, N = 20) responses are very similar. If both stripes get displaced (‘Both’, N = 21), the average torque trace is comparable to that of the syn-directional responses to the single-bar displacements. (D) Response frequencies. (E) Response latencies. Error bars are SEMs (*P < 0.05, **P < 0.01, ***P < 0.001).

One black vertical stripe (height = 90°; width = 18°; azimuth: ψ_0_ = + or—45°) is displaced front-to-back (Δψ = 30° at a velocity of v = 150°/s). At the torque meter in the center of the light-guide arena (Fig 1A and 1B), the fly responds to this stimulus in one of three ways. Most often it generates a fast increase of yaw-torque with the same polarity as the motion stimulus (Fig 1C; ‘Single’, syn-directional response). Under closed-loop conditions [18] this response would immediately reset the bar to about its initial position correcting for the disturbance in flight direction. Without visual feedback the fly, remarkably, may also respond with the opposite polarity, turning away from the stripe (Fig 1C; anti-directional response). Often the fly shows no response (nr) at all (Fig 1D for response frequencies). For anti-directional responses the response latency is shorter (Fig 1E) and also the phasic torque response itself is shorter (Fig 1C). While in free flight syn-directional responses would serve the fly to stabilize its orientation in space, the fast anti-directional responses might be attempts to escape the attack of a predator.

Right after the fast front-to-back displacement the stripe is slowly (v = 20°/s) moved back to its initial position (Fig 1B). After a syn-directional response yaw torque returns during that phase of 1.5s to the level from which it had started. After an anti-directional response in the first phase the fly generates a weak syn-directional response to the slow back-to-front motion and returns to base line only after the motion has stopped (Fig 1C). If the fly is confronted with two stripes at ψ_0_ = + and—45° and only one of them is displaced ('One of two' in Fig 1C), the fly's responses are much the same as with a single stripe.

**Fig 2:**
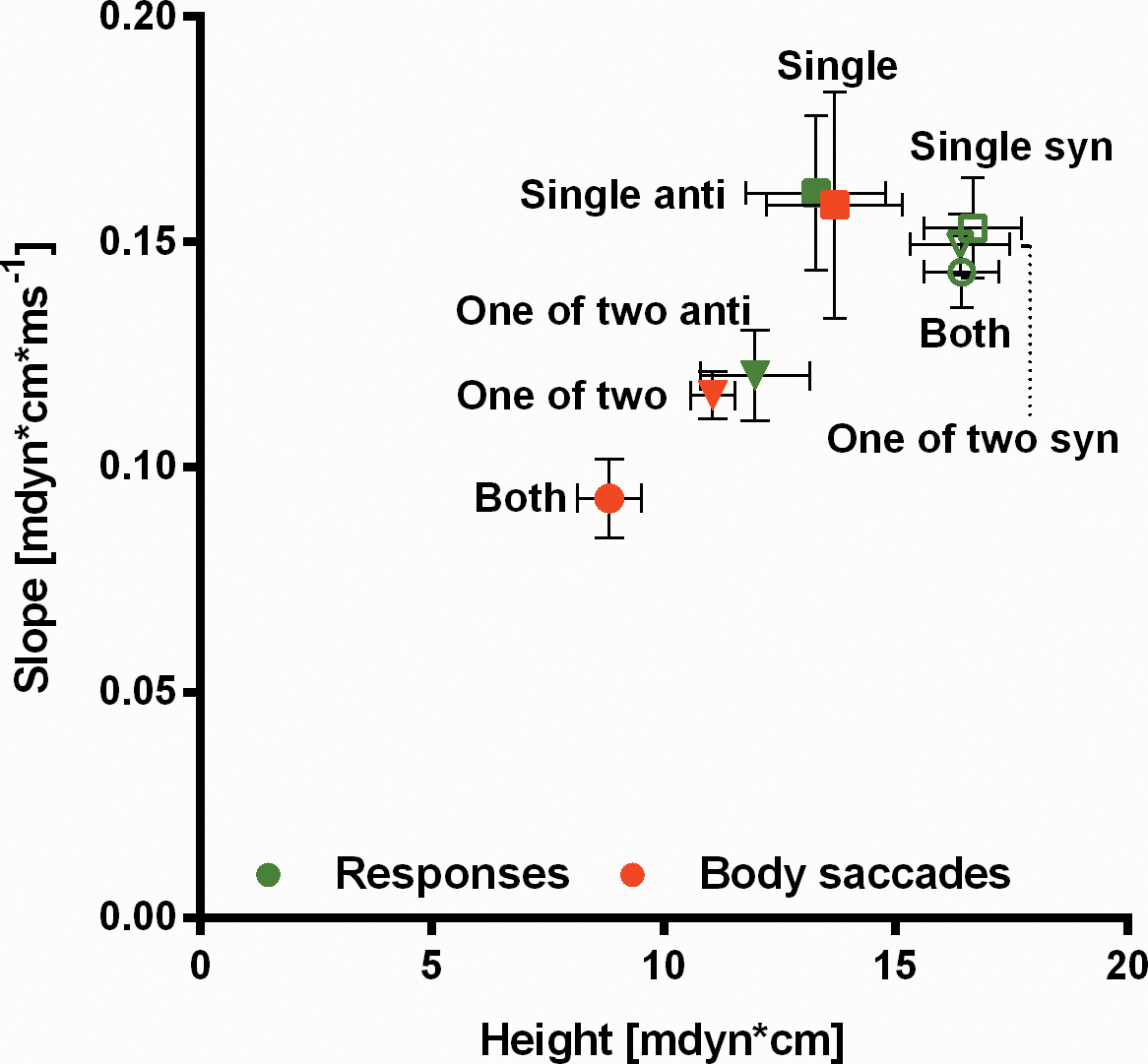
Slope of the rising phase and height of the torque responses (green) and spontaneous body saccades during ITIs (red). For the experimental conditions ‘Single’ (N = 21) and ‘One of two’ (N = 20) data are split into ‘syn’ and ‘anti’ with regard to the response polarity. For the experimental condition 'Both' no such differentiation can be made. Syn-directional responses have a similar shape as the ones generated when both stripes are displaced ('Both'; N = 21). Body saccades differ in slope and height in the three experimental conditions ('Single', 'One-of-two', 'Both'). Anti-directional responses resemble body saccades.

In between test trials when the stripes are not moving the fly occasionally generates spontaneous body saccades [18]. Interestingly, these can be well distinguished from the syn-directional responses but resemble in their dynamics the anti-directional ones. The dynamics differ between the single-stripe and 'One-of-two' experiments (Fig 2) indicating that they are influenced by other stimulus parameters besides visual motion.

### Simultaneous displacement of two stripes

To characterize SVA we simultaneously displace two stripes front-to-back, one at ψ_0_ = +45°, the other at ψ_0_ = -45° in front of the fly, and shift them slowly back to their initial positions (Fig 1C, ‘Both’). The responses have the longer latency (Fig 1E) and longer duration of the syn-directional responses above as well as the larger amplitude (Fig 1C). Judged by the response latency, the putative escape responses do not occur under these conditions. With both stripes being displaced simultaneously the overall response frequency might be expected to be the sum of the response frequencies of two single-stripe experiments. On the other hand, as the vector sum of the two movements is close to zero, the response frequency might also be expected to drop to zero. Both assumptions turn out to be wrong. The response frequency for each stripe is lower than the frequency of syn-directional responses in the single-stripe experiments and the overall frequency is only slightly higher (Fig 1D). In other words, the simultaneous displacement of the second stripe reduces the frequency of responses to the first one. As discussed already by Sareen [16], this suppression alone could be explained by mutual inhibition between the central pattern generators (CPGs) for cw and ccw flight turns. However, the authors describe a cueing effect which allows to direct the FoA to one or the other side. This favors SVA over mutual inhibition. In the following experiments we further characterize SVA in flies measuring the attention span, i.e. the time the FoA remains at the location to which it had been shifted. An attention span would not be expected for the mutual-inhibition concept.

### Is the choice of response polarity influenced by the previous choice?

**Fig 3:**
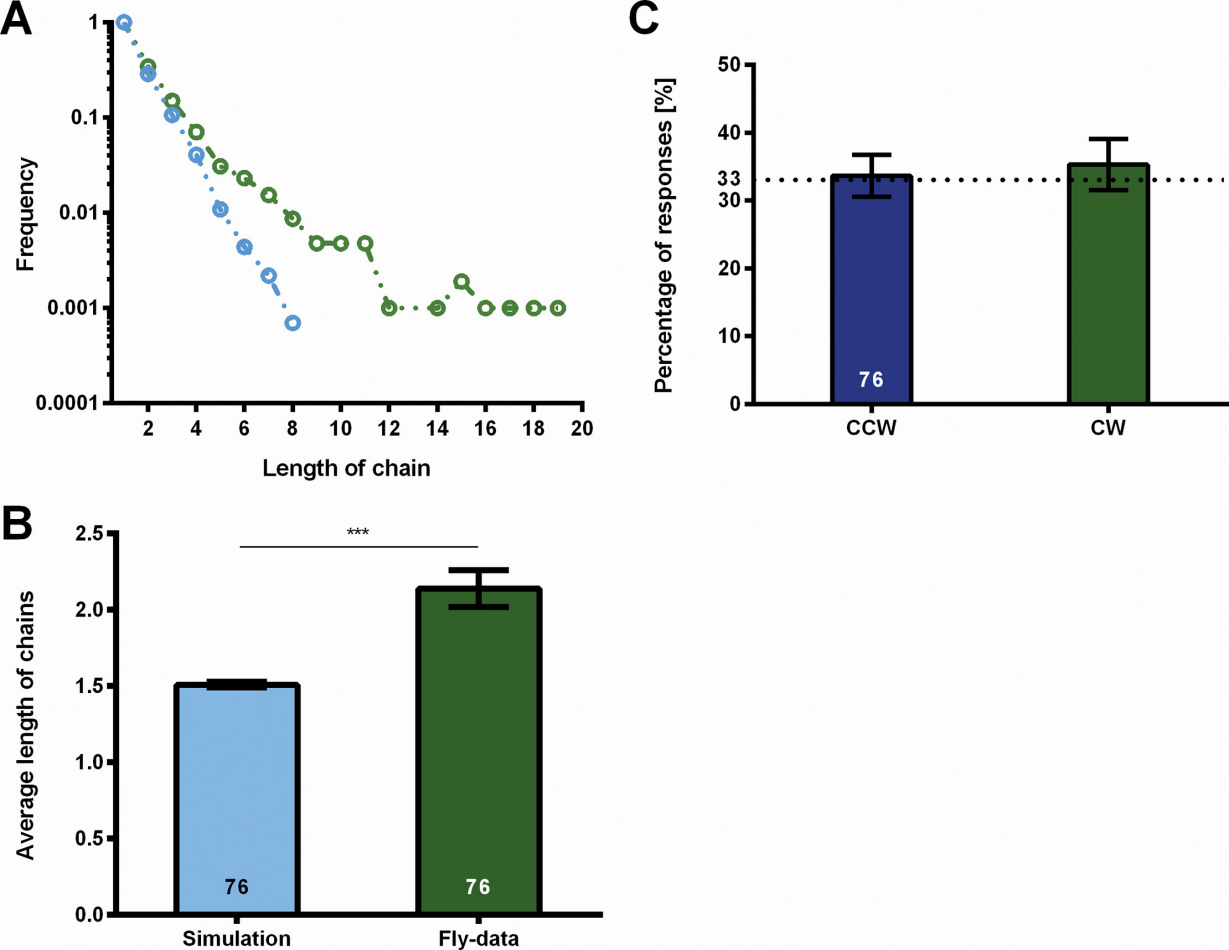
Response frequencies and chain lengths. Chains are consecutive identical responses. (A) Frequencies of chain lengths. The green line represents frequencies of chains of certain lengths. The blue line shows the chain length frequencies calculated from the mean response frequencies shown in (B) assuming random choices. (C) Average lengths of chains. Calculated mean chain length differs significantly from measured one. Error bars are SEMs (*P < 0.05, **P < 0.01, ***P < 0.001).

In a series of 60 displacements with an inter-trial interval (ITI) of 1s, we measure for each test the response polarity. Chains of consecutive responses with the same polarity (e.g. cw-cw-cw…), for the sake of brevity just called chains, are extracted from the data as a measure of the constancy of the fly's response polarity over time. With increasing length the frequency of chains decreases (Fig 3A). The average chain length for an ITI of 1s is cl = 2.14 ±0.12 (Fig 3B, fly data). If the response polarity is exclusively determined by chance, the distribution of chain-lengths generated by the flies can be calculated from the mean frequencies of cw and ccw responses (rf_ccw_ = 0.33; rf_cw_ = 0.33; see Fig 3C). A pronounced difference between fly-data and the data calculated from the mean frequencies is found. Also, the average chain length calculated from the mean frequencies (cl = 1.49 ±0.01; Fig 3B, simulation) is significantly lower than the chain length measured directly. Thus, the choice of response polarity cannot be exclusively random, but must follow a mechanism that favors the formation of chains.

### Chain length is affected by different mechanisms

There are at least two possible underlying mechanisms that could explain these differences. One is the attention span: The response could be influenced by the preceding one such that the likelihood for the same polarity would be increased by a certain factor (dwelling factor, *df*). As a second effect, each fly could have an individual preference for responses towards one side (sidedness).

**Fig 4:**
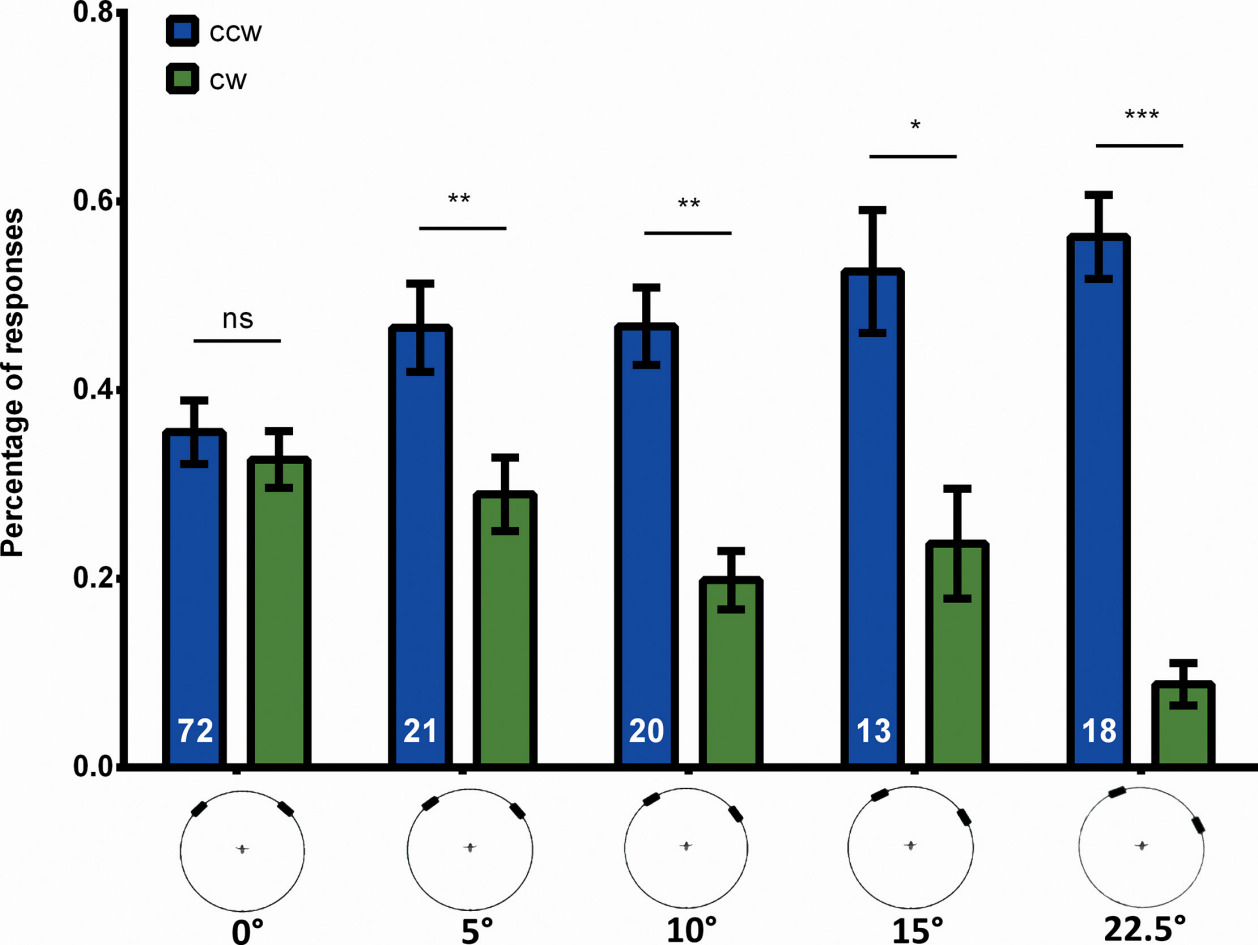
Effects of externally caused sidedness on response frequency. When two stripes are simultaneously displaced at ψ0 = ±45°, flies respond to either stripe equally often. If the longitudinal axis of the fly is slightly shifted with respect to the stripes, the fly favours the stripe that is more in front. Error bars are SEMs (*P < 0.05, **P < 0.01, ***P < 0.001).

To assess the attention span we need to control sidedness. Already a minute deviation of the longitudinal axis of the fly from the line of symmetry creates a preference for the stripe closer to the midline (Fig 4). Such a dependence of response frequencies on the azimuth of the stripes already becomes apparent in the work of Sareen [19]. Fortunately, with 60 test trials the experiment allows us to separately calculate the sidedness of each fly measuring the overall difference between cw and ccw responses (see Methods). Being interested in the dynamics of the response polarity we eliminate flies with a large sidedness (see Methods) from the evaluation and, for the remaining flies calculate the *df* for each fly as the mean of the *dfs* for cw and ccw responses.

**Fig 5:**
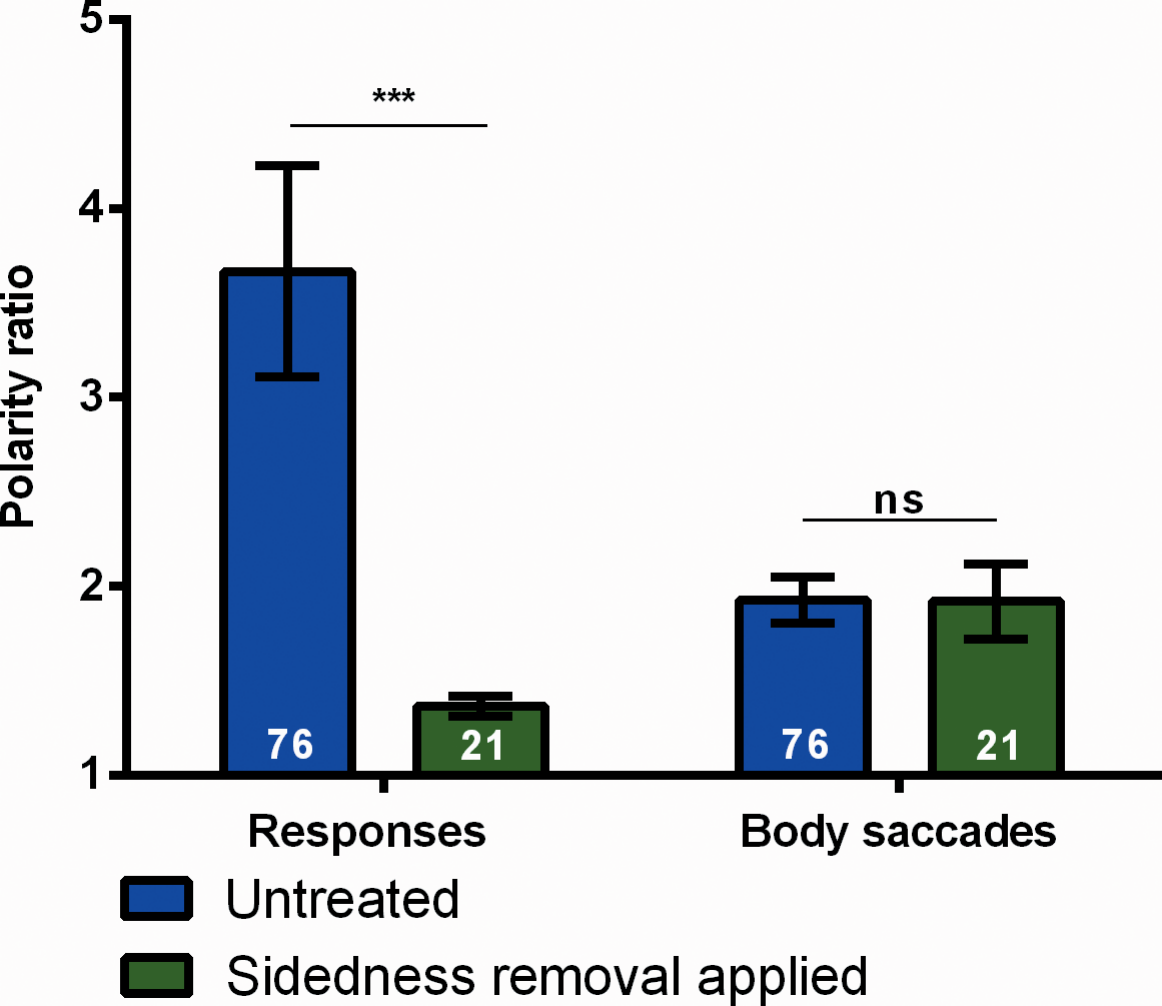
Polarity ratio (cw / ccw) of responses and body saccades (during ITIs). For each fly a polarity ratio is calculated as the number of spikes of the more frequent polarity divided by the number of spikes of the less frequent polarity. Numbers in the bars indicate N of flies. Whereas the polarity ratio of responses is susceptible to the removal of sidedness, that of body saccades is not (see Methods for details). Error bars are SEMs over flies (*P < 0.05, **P < 0.01, ***P < 0.001).

In contrast to responses, spontaneous body saccades, which occur during ITIs are not subject to this kind of sidedness. Eliminating flies with a high sidedness from the evaluation does not significantly change the polarity ratio for body saccades, suggesting two different behaviors (Fig 5).

### Duration of attention span

**Fig 6:**
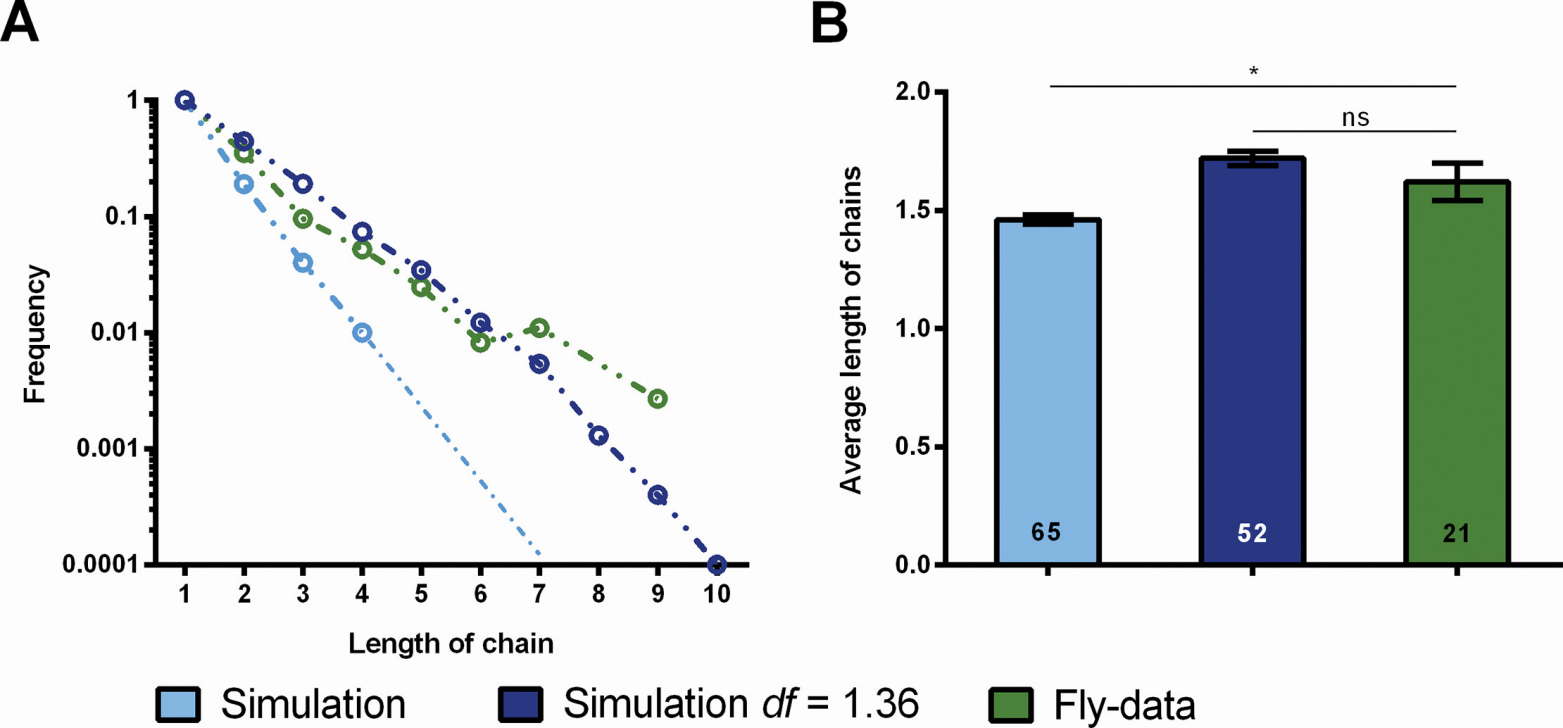
Chain lengths after removal of sidedness. (A) Chain length frequencies. After removal of sidedness calculated chain lengths (light blue) still differ significantly from measured ones (green). This difference is attributed to dwelling (attention span at the FoA). When a dwelling factor of df = 1.36 is used to calculate chain length frequencies, simulation and fly-data give the best fit (dark blue). (B) Average chain lengths. Difference between calculation without dwelling (light blue) and fly data (green) is significant. Error bars are SEMs (*P < 0.05, **P < 0.01, ***P < 0.001).

With sidedness largely removed from the further evaluation of the data we find a mean *df* = 1.36 for CantonS wild-type. The *df* indicates a by 36% increased probability of a repetition of the preceding response polarity as compared to the initial value (e.g. after a response towards the left stripe the initial probabilities rf_ccw_ = rf_cw_ = 0.33 are set to rf_ccw_ = 0.448 and rf_cw_ = 0.276 for the next displacement). The higher probabilities of longer chains in the fly data (after correction for sidedness, Fig 6A, ‘Fly-data’ and ‘Simulation’) are thus explained by a tendency of the fly to repeat the previous response. Using for the simulation of chain length distribution the fly data corrected for sidedness, one minimizes the difference between this simulation and the observed chain length distribution (Fig 6A, ‘Fly-data’ and ‘Simulation *df* ' = 1.36).

As pointed out in the introduction, we take the cueing experiments to show that the fly has its FoA at the location of the stripe to which it responds. This allows us to interpret the fly's tendency to repeat the previous response as a dwelling at the location to which the FoA had been shifted. In other words, SVA has an attention span.

**Fig 7:**
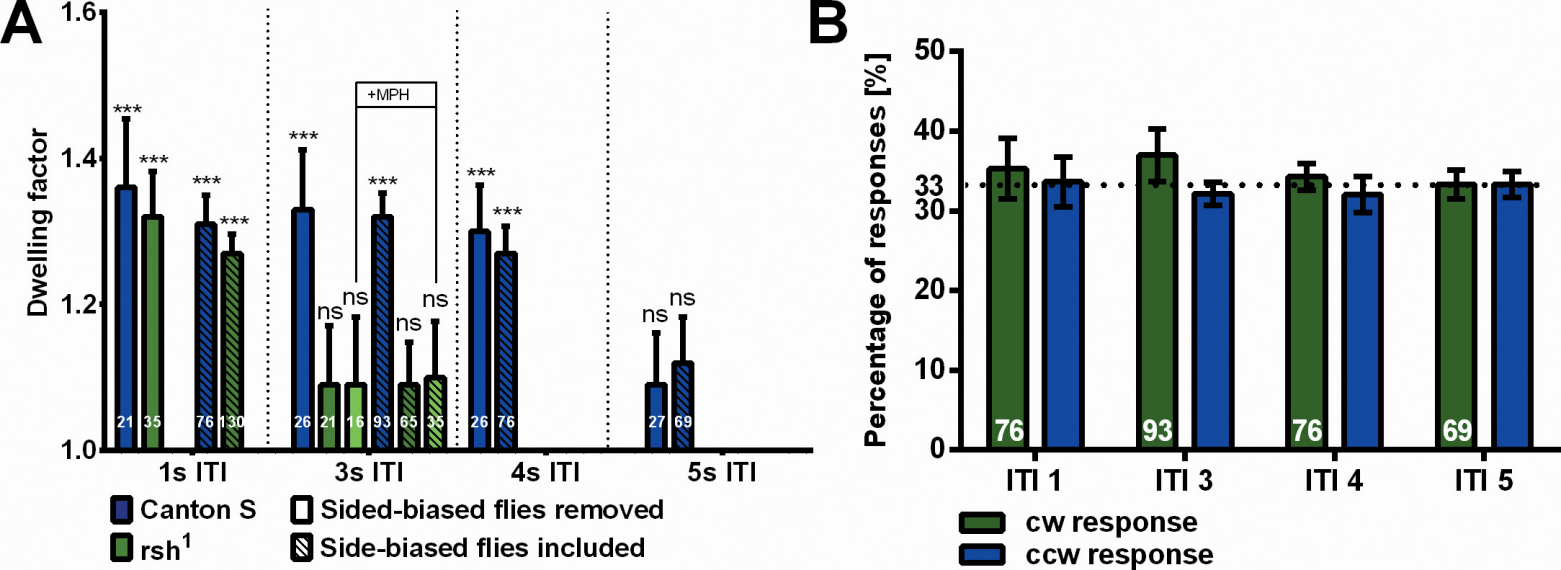
Temporal limitation of dwelling. (A) When the ITI is prolonged, the estimated dwelling factor does not decrease significantly in CantonS flies during the first 4s. However, the attention span lasts less than 5s. In the mutant rsh^1^ flies show wild-type like dwelling for an ITI of 1s, but the attention span lasts less than 3s. The same results are obtained, if the selection procedure (see Methods) for unbiased flies is not applied to the data. (B) For all four sets of CantonS experiments (ITI: 1, 3, 4 and 5s) the response polarity distribution is balanced for left and right responses. In all sets flies flew robustly and produced comparable patterns of responses. It is thus unlikely that differences in the *behaviour* of different batches of flies cause the observed differences.

To measure the duration of the attention span we prolong the inter-trial interval (ITI). In the series of 60 trials the longer intermissions have no substantial effect on the dynamics and frequency of the responses. Computing the *df* for each condition we find a non-significant decrease for ITIs of 1, 3 and 4s (Fig 7; *df*_1s_ = 1.36; *df*_4s_ = 1.30). For an ITI of 5s a significantly smaller dwelling factor (*df*_5s_ = 1.09) is observed. Apparently, after 4 to 5s the dwelling effect wanes and the probabilities of the response polarity return to chance level. The attention span of SVA under the conditions of the experiment is 4 to 5s. The filtering steps applied to eliminate the influence of externally caused sidedness do not interfere with this finding. When we do not select for flies that express a minimum of sidedness, we still arrive at the same result. Also, the response rates and response polarity distributions of all sets of tested flies are almost the same (Fig 7B). This finding excludes the possibility that the observed reduction of the dwelling-factor after 5s might be due to poor flight performance of the tested flies.

### Attention span in *radish*^*1*^ mutant flies

Recently, flies with a mutation in the *radish* (*rsh*) gene were shown to be defective in a behavioral property that was called attention-like [20]. Flies walk through a maze with eight consecutive right / left choices. Underneath the maze they see a grating moving perpendicular to their overall walking direction. Wild-type flies are influenced in their choice behavior by the motion. They tend to follow the direction of motion. Mutant flies are less affected. Their choices are closer to random. On the basis of a more detailed behavioral analysis the authors hypothesize, that something like attention might be involved in the wild-type behavior and that this property would be affected in the mutant.

**Fig 8:**
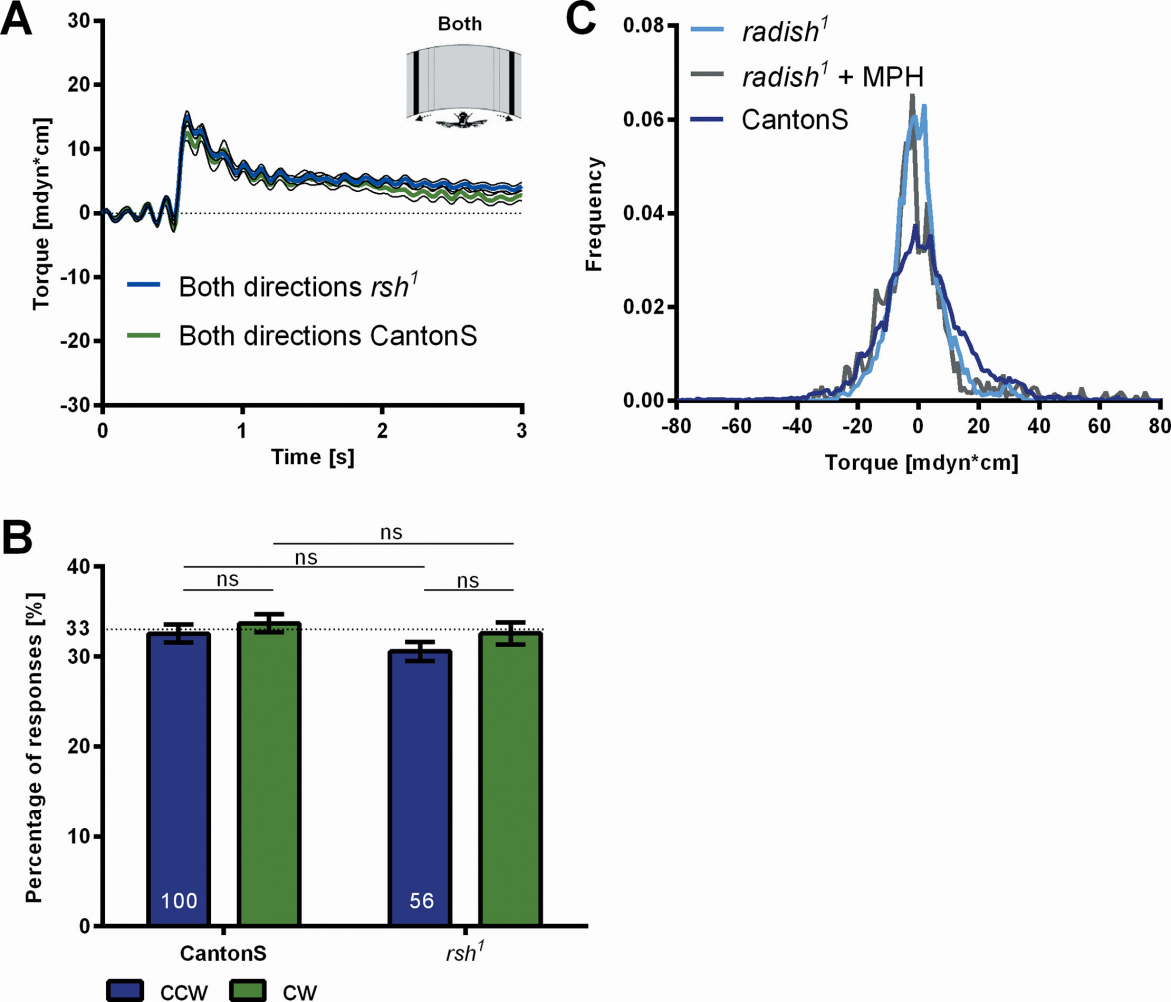
Flight behavior of mutant *rsh*^*1*^. (A) Average time traces of CantonS and *radish*^*1*^ torque responses to the displacement of both stripes (‘Both’, N = 21 and N = 35). Responses of CantonS have a slightly smaller amplitude than those of *radish*^*1*^. (B) Response frequencies of *radish*^*1*^ and CantonS show no significant differences. (C) Normalized histograms of yaw torque generated by the flies within 1s intervals between test trials. Torque modulations are slightly reduced in *radish*^*1*^ as compared to CantonS (N = 35 and N = 100). The reduction of torque modulation remains unaltered by MPH treatment of *radish*^*1*^ flies (N = 16). Error bars are SEMs (*P < 0.05, **P < 0.01, ***P < 0.001).

One wonders, whether the assumed attention in maze walking and the attention we measure in stationary flight at the torque meter might be related. Would *rsh*^*1*^ flies also be disturbed in SVA in stationary flight? The dynamics of the responses to the displacements of the two stripes and the overall response frequencies are normal in the mutant (Fig 8A and 8B). With a pause of 1s between displacements no substantial difference in dwelling (*df* = 1.32) is observed compared to CantonS wild type. So far, SVA in the *rsh*^*1*^ mutant seems to be normal. To measure the attention span of *rsh*^*1*^ we increase the ITI. Interestingly, at an ITI of 3s *rsh*^*1*^ flies no longer show significant dwelling (*df* = 1.09; Fig 7). These results would be in line with the hypothesis that also in maze walking the attention span might be reduced in the mutant and that the two kinds of visual attention involve the same mechanism generating an attention span. This interpretation, however, is rejected by the following experiment.

In their study van Swinderen and Brembs [20] hypothesize that the defect of the *rsh*^*1*^ mutant in maze walking might be due to some kind of hyperactivity relating it to attention-deficit-hyperactivity syndrome (ADHS) in humans. They strengthen this assumption by the additional finding that methylphenidate (MPH), a drug commonly used to treat ADHS in humans, also 'cures' the maze walking defect in *rsh*^*1*^ flies. For SVA in the present experiments MPH does not revert the attention span of *rsh*^*1*^ to that of wild-type CantonS (Fig 7). This result argues that the attention spans in the maze and in SVA are not the same and that the *rsh* gene is differently involved in the two. The *rsh* gene must be involved in a variety of behavioral processes. For instance, the yaw torque modulation of *rsh*^*1*^ in tethered flight is narrower than that of CantonS wild type (Fig 8C). This may be the reason why the height of its responses in the test appears to be even slightly larger (Fig 8A). (See the Discussion below for further considerations regarding different kinds of visual attention.)

### Focus of attention is shifted independently of yaw-torque

**Fig 9:**
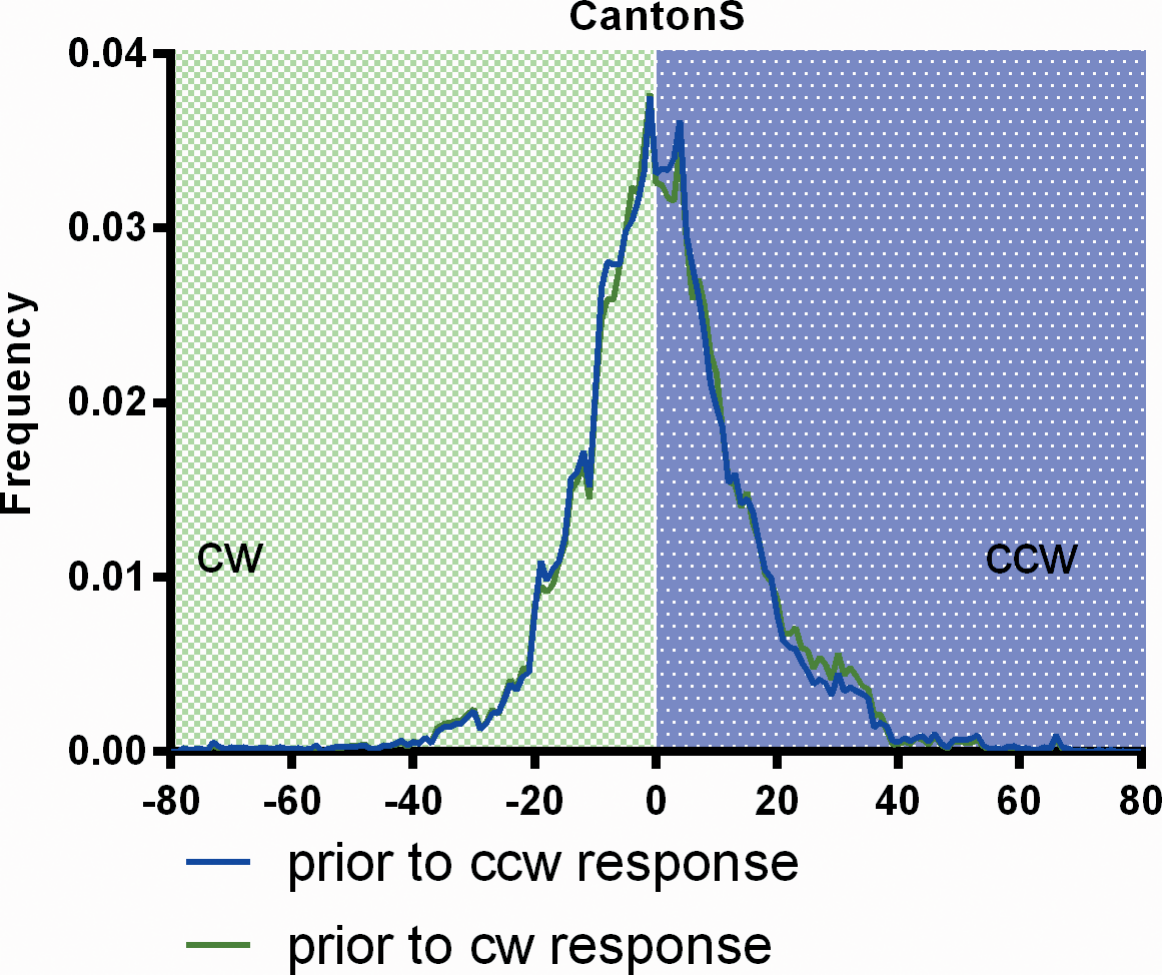
No influence of yaw torque on response polarity. Histograms of CantonS yaw torque generated within 1s before stripes where displaced. For each measurement the data were categorized according to the polarity of the response (cw or ccw; N = 100).

In a visual learning task at the torque meter Tang [21] presented suggestive evidence that yaw torque and the position of the FoA might be connected. If this were the case in our study, the dynamics of the response polarity would not only reflect the dynamics of the FoA, but also that of the central pattern generators determining flight direction. To address this issue we analyze the yaw torque generated by the flies during 1s before the onset of a displacement and categorize it with regard to the subsequent response polarity (‘cw’ or ‘ccw’). If the preceding torque level would influence the following response polarity, a difference in the torque level before the response should be seen between the two groups. No such difference is observed, even if the data of all experiments are pooled (Fig 9). Thus, in our paradigm flies are able to shift their FoA independently of yaw torque.

### No 'inhibition of return'

**Fig 10:**
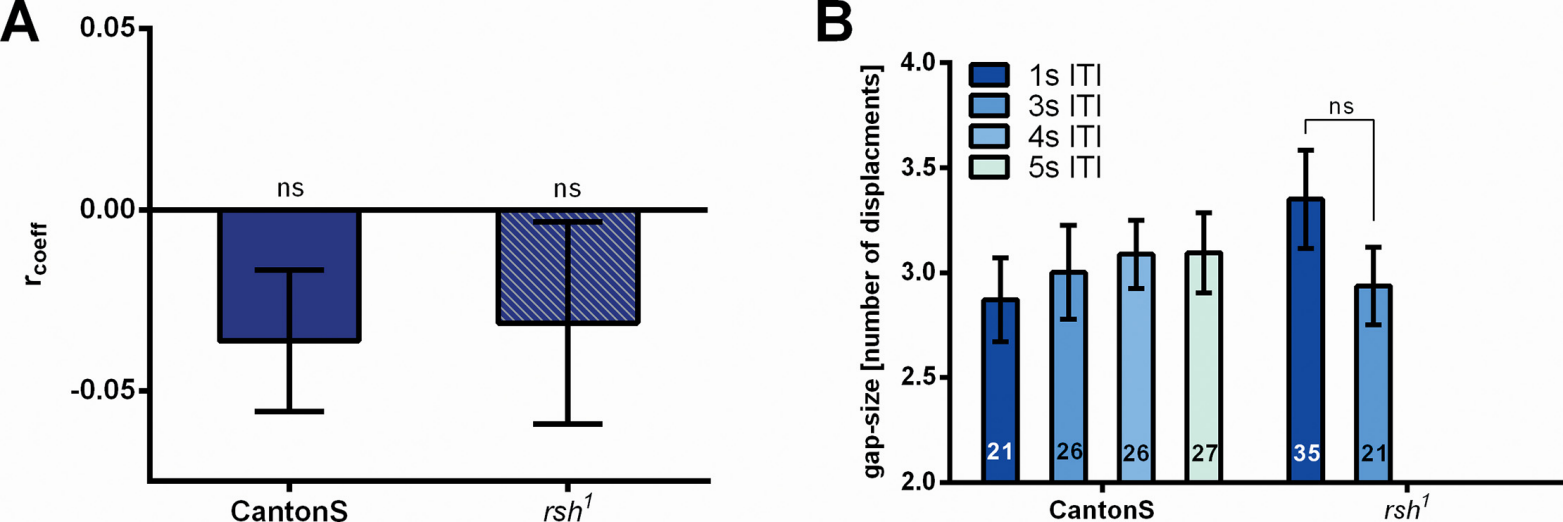
Attention span is not explained by inhibition of return. (A) Neither in CantonS nor in *rsh*^*1*^ (N = 21 and N = 35) a correlation is found between chain length on one side and subsequent number of responses to the other side (and no responses). (B) After a chain of responses to one side, the number of displacements it takes the fly to return with its response polarity to that side, does not depend upon duration of ITIs. Error bars are SEMs (*P < 0.05, **P < 0.01, ***P < 0.001).

How is the attention span generated? From mammalian covert attention it is known that reaction times towards a target in a recently examined area are increased as opposed to a target at a new location [22–24]. Likewise, in our case the FoA might stay on the chosen side, because it is repelled by the side where it had been before. In our series of 60 test trials such an inhibition might show. For instance, if inhibition would gradually build up with the time the FoA stays on one side, chain length (e.g. chain length_cw_) might be positively correlated with the size of the gap (e.g. gap_cw_ = response type ≠ cw) until the next chain of responses to the same side (cw) would be initiated. Neither CantonS nor *rsh*^*1*^ flies show such a correlation (Fig 10A). If the duration of inhibition had a fixed value rather than depending on the time the FoA dwelled at the previous location, one would expect to the size of the gaps between chains on the same side to depend upon the duration of the ITIs. Again, neither for CantonS nor *rsh*^*1*^ flies such an effect is observed (Fig 10B). In the series of experiments with different durations of ITIs, the found average pauses for all ITIs of 3 test trials (i.e. the duration of IoR) would last from 8.1s (ITI_1_ = 1s) to up to 20.1s (ITI_5_ = 5s). These results do not support the hypothesis that inhibition of return is a major part of a mechanism for the attention span.

## Discussion

With the recording of an attention span we consolidate the interpretation of this set of behavioral symptoms as selective visual attention (SVA). Taking advantage of the fly's ability to shift the FoA endogenously we measure that the fly keeps the FoA for several seconds at the location to which it had shifted it in the previous test trial. Trapped at the torque meter flies presumably are in a state of arousal or stress. How long the attention span would last under more relaxed and more natural conditions such as free flight, walking in a cluttered surround or during rest, we do not know.

The FoA remains at the location where a few seconds earlier a stimulus had elicited the fly's behavior. However, the behavior has been a failure since it did not generate the expected feedback from the visual surround. Why it might be adaptive to keep the FoA there is a matter of speculation. Even under natural conditions a fly detecting motion at a certain location of the visual field would need to understand its behavioral relevance in order to generate an adaptive response. An answer or at least further information on the problem is more likely to be found at the same location than somewhere else. Therefore the fly sticks to this region and further investigates it or at least waits whether more is going to happen there. The long duration (> 4s) might be favored by the few distractors in the fly's uniform surround of the light-guide arena. Nevertheless, the lack of distractors or visual feedback of torque modulations do not lead to a habituation of the response rate. The rate of no responses does not increase during the course of the experiment.

Some years ago van Swinderen [25] identified a hypothetical electrophysiological correlate of an attention span in the *Drosophila* brain. He measured the oscillation amplitude within a certain frequency range of a local field potential (LFP). The fly was tethered and two visual patterns where rotated around the fly at 0.33Hz, such that each pattern moved through the frontal visual half-field in 1.5s. If a new pattern was introduced into the arena, the LFP was larger if this pattern was in the frontal part. This effect was interpreted as a 'preference' for the novel pattern. The preference was taken to indicate, to which of the two patterns the fly attended. The attention span was then defined as the average number of cycles for which the LFP continuously showed the same preference.

It is not beyond doubt that the increased LFP in response to a novel pattern reflects an attentional mechanism. If it does, the requirements for this visual attention are distinct from those of SVA. There is no FoA that must be shifted in the visual field to give an attention span. They are also distinct from those of visual attention in maze walking as the latter does not involve pattern recognition and novelty choice. Different requirements might recruit different forms of visual attention (e.g. feature-based, object-based or spatial attention) in *Drosophila*. Each one will have to be characterized separately before one can tell how they might be related.

Except for its duration we know close to nothing about the attention span in SVA. Does the shape and size of the moving bar matter? Is it continuous? We only know that the fly has the tendency to choose the same response polarity as in the previous test if the interval between the two tests is less than 5s. In this part of the visual field the fly is more sensitive to the test stimulus than at the mirror-symmetrical location on the other side. We infer that the FoA has remained at the same location where it had been before. To further strengthen this interpretation it would be important to find modifications of visual processing in other parts of the visual field and show that they persist at their locations as well. Comparing the dynamics of the responses in the test trials to those of the single-stripe displacements, one notices that in the slow return phase of the stripes yaw torque is also affected by the motion of the stripe on the non-attended side (Fig 1C). Whether this implies that the FoA is transiently shifted to the other side during the return phase or that the sensitivity to slow back-to-front motion is especially high outside the FoA, is not known.

Formally, the attention span is an aftereffect of the fly's action selection process in the last test trial. Is the aftereffect of an external cueing event as described by Sareen [10] the same as the attention span observed here? Clearly not. The cue signals a property of the outside world which might well be still present a few seconds after it has disappeared from sight. The attention span is a state of the animal which it maintains despite (or because of) the failure of the last behavioral action. There is no cueing involved in the present experiments. This does not rule out that in the two aftereffects similar processes at the neuronal and/or molecular level might be involved. The *rsh* gene marks such a case. It will be important to find further mechanistic components of SVA.

This work is a first attempt to characterize the attention span in covert SVA in flies. SVA is a fundamental property of visual perception presumably going on most of the time in the normal wake life of flies and other animals. In humans covert SVA is mostly transient. It directs eye and head movements before overt SVA takes over. In *Drosophila* overt SVA is yet to be described.

## Materials & Methods

### Flies

Flies were cultured at 25°C on standard medium with 60% relative humidity on a 12h light / dark cycle. Wild-type flies were of the CantonS (CS) strain and the *radish*^*1*^ mutant was obtained from Josh Dubnau (Cold Spring Harbor Laboratory, Cold Spring Harbor, NY). For pharmacological rescue experiments 2 days old *radish*^*1*^ flies were put on 10ml of regular fly food that additionally contained 5mg of methylphenidate hydrochloride (Sigma) and blue food dye for 14h. Intake of the food was verified by checking for a blue color of the flies’ abdomen. For tethering 2 to 4 days old female flies were anesthetized by cooling and glued with dental composite (ESPE Sinfony™ DO3, 3M, Neuss, Germany) to a triangle-shaped wire-hook made of copper (Ø = 0.05mm) using a micro-manipulator. The tip of the holder was positioned between the fly’s head and thorax to prevent independent motion of the two body parts. The glue was then polymerized using a blue LED light source (10s pulse, distance < 5mm) and flies were kept in single vials with access to water for a minimum of 2h.

### Setup

Visual stimuli were presented in a cylindrical arena using fiber-optics. 32 x 180 single light-guides connected a rectangular frontal plate with the inner surface of the arena (Ø = 90mm, h = 90mm). Computer-generated visual stimuli were projected (BenQ W770ST, 120Hz) onto the frontal plate and via the light-guides to the inner surface of the arena. The arena covered 360° x +/-45° of the fly's visual sphere. To prevent light from outside the arena to reach the eyes of the fly, the floor of the arena was covered with black cardboard and the complete setup was positioned in a small dark chamber. Position, timing and geometrical properties of the visual stimuli were controlled and updated by custom-made software written in VB.NET at 300Hz. The fly glued to the wire-hook was attached via a clamp to the torque meter and centered in the arena (for further details see [26]). To align the fly to the visual stimuli, the center line of the arena was marked during mounting with a red laser beam and flies were thoroughly aligned to it.

### Stimulus conditions

Two black 18° wide stripes were presented on a white background. Their centers were located in the fronto-lateral visual field of the fly at ψ_0_ = ±45°. Stripes were displaced by Δψ = 30° with fast front-to-back motion of v = 150°/s, followed by a slow reset to their initial position at v = 20°/s. The experiment consisted of a series of 60 displacements. During the inter-trial interval (ITI) the stripes remained stationary at the ψ_0_ position for 1s (unless stated otherwise). In most of the cases flies responded to the front-to-back motion with a phasic yaw torque modulation. A response was scored when the modulation exceeded the range between maximum and minimum torque values recorded within 0.5s prior to displacement by more than 60% within 0.5s after onset of the displacement. Left (counter clockwise; ccw) and right (clockwise; cw) responses as seen from the position of the fly were scored separately. No responses (nr) were scored, if there was no sufficiently large yaw torque modulation (see above).

### Data evaluation

Yaw-torque was stored on the controlling computer’s hard disk at 100Hz and later evaluated using custom-made software (VB.NET). To measure the attention span, the sidedness, i.e. the difference of the left and right mean response frequencies (rf_ccw_; rf_cw_) needed to be small. We therefore excluded some flies from the data evaluation. Three exclusion criteria were used: (1) An asymmetry index (AI) was calculated for each fly as AI = |rf_ccw_-rf_cw_| / (rf_ccw_+rf_cw_). Flies with AI > 0.3 were not used for further evaluation. (2) Mean chain lengths of consecutive identical responses (cl_ccw_; cl_cw_) were derived from the data separately. The absolute difference of mean chain lengths (AD_cl_ = |cl_ccw_—cl_cw_|) was another exclusion criterion. Only flies with AD_cl_ < 0.6 were further evaluated. (3) Flies with an overall low response rate (RR < 0.6) were also excluded from the evaluation. The thresholds for exclusion were set such that by visual examination the long tails of the respective distributions were cut away. These filtering steps yielded flies with a balanced number of left and right responses (rf_ccw_; rf_cw_), with the pooled response frequencies still matching those of the unfiltered data-set.

To detect dwelling, the fly data were compared to simulated data-sets with various dwelling factors (*df*, ranging from 1 (≙ no dwelling) to 2 with an increment of 0.01). Each data-set was obtained from 1000 repetitions of 60 random choices among the three response types. The basic probability for each response type was set to 0.33 to match the overall response probabilities measured in flies. Comparisons were carried out separately for both response polarities (ccw and cw) and the *df* for each fly was then calculated as the mean of the *df*_*ccw*_ and *df*_*cw*_ that had each resulted in the best fit of simulated and fly-data using the Gaussian least squares method. Remaining sidedness, by definition, could only have an effect on *df*_*ccw*_ or *df*_*cw*_ and would increase the average length of chains on a side. But any sidedness causing an increase of the *df* on one side will at the same time decrease the *df* on the opposite side by the same amount. Thus the applied averaging of *df*_*ccw*_ and *df*_*cw*_ removes sidedness, leaving only the effects of dwelling.

### Statistical analysis

All data were tested for normal distribution using a Kolmogorov-Smirnov test. Since the data Fig 3B, Fig 5, Fig 6B, Fig 7A, Fig 8B, Fig 10A and 10B were normally distributed, a one-sample t test was used to compare values with a random value and a two-sample t test was used to compare values with each other. Bonferroni corrections were used for multiple comparisons. Because not all data in Fig 1D, 1E and Fig 4 were normally distributed, Wilcoxon Matched Pairs test for dependent pairwise comparisons was used to test responses to one side against responses to the other side and Wilcoxon Signed Rank test was used to compare values with a specific value (* = p < 0.05, ** = p < 0.01, or *** = p < 0.001).
